# Supplementary material for: Hsp multichaperone complex buffers pathologically modified Tau
Source: Nat Commun. 2022 Jun 27;13:3668. doi: 10.1038/s41467-022-31396-z (PMC9237115; doi:10.1038/s41467-022-31396-z)
Supplement: Supplementary file 1 — Supplementary Information [file 41467_2022_31396_MOESM1_ESM.pdf]

Supplementary Information

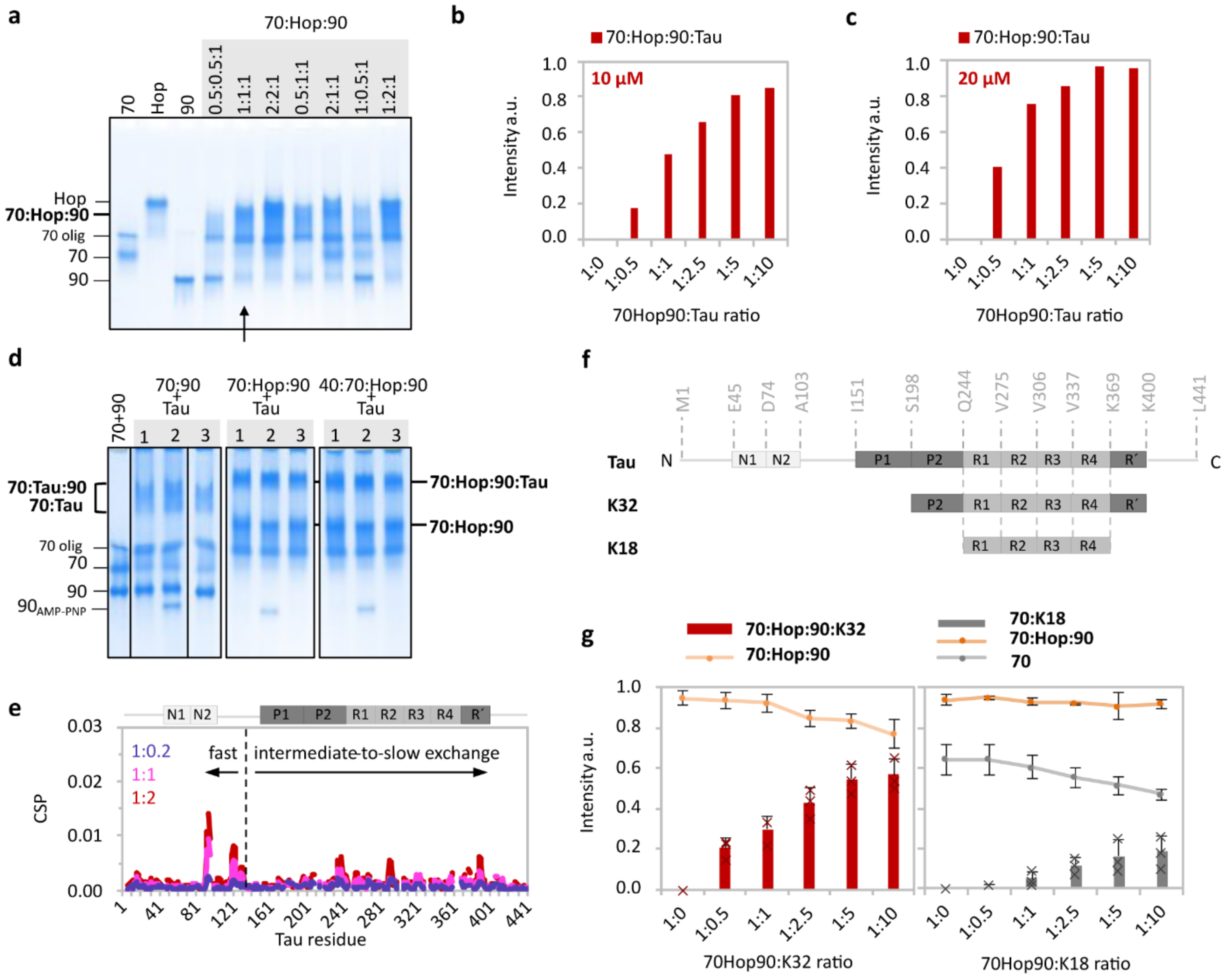

Supplementary Fig. 1. Tau interacts with the Hsp70/Hsp90 chaperone machinery (related to Fig. 1). A) Native PAGE analysis demonstrating Hsp70:Hop:Hsp90 complex formation when mixing the proteins in a molar ratio of 1:1:1 (indicated by the black arrow). Hsp70 and Hsp90 are abbreviated as “70” and “90”, respectively. For Hsp70, a fraction of the protein was oligomeric (labelled as “70 olig”). B-C) Quantitative analysis of the formation of the Hsp70:Hop:Hsp90:Tau complex at different absolute chaperone concentrations of 10 µM (b) and 20 µM (c), from one representative gel per concentration. D) Native PAGE analysis of the Hsp70:Hsp90 (1:1) and Hsp70:Hop:Hsp90 interaction (1:1:1) with or without the substrate Tau (:5) or Hsp40 (:0.4; abbreviated as “40”) in the absence and presence of nucleotides (1/no nucleotide, 2/+AMP-PNP, 3/+ADP). E) NMR chemical shift perturbations observed in (Fig. 1e) using Tau:machinery molar ratios of 1:0.2 (purple), 1:1 (pink) and 1:2 (red). I_0 are the peak intensities of unbound Tau (grey spectrum in Fig. 1e). The black dotted line marks the distinction between fast and intermediate-to-slow exchange referring to low and high affinity, respectively. Tau domains are indicated on top. F) Domain organization of Tau, K32 and K18. G) Quantitative analysis of the band intensities from native PAGE demonstrates the interaction of the K32 construct (left panel) with the Hsp70/Hsp90 machinery, whereas the repeat region alone (K18, right panel) does only bind to Hsp70. Data are presented as mean +/- standard deviation (SD) from three independent experiments.

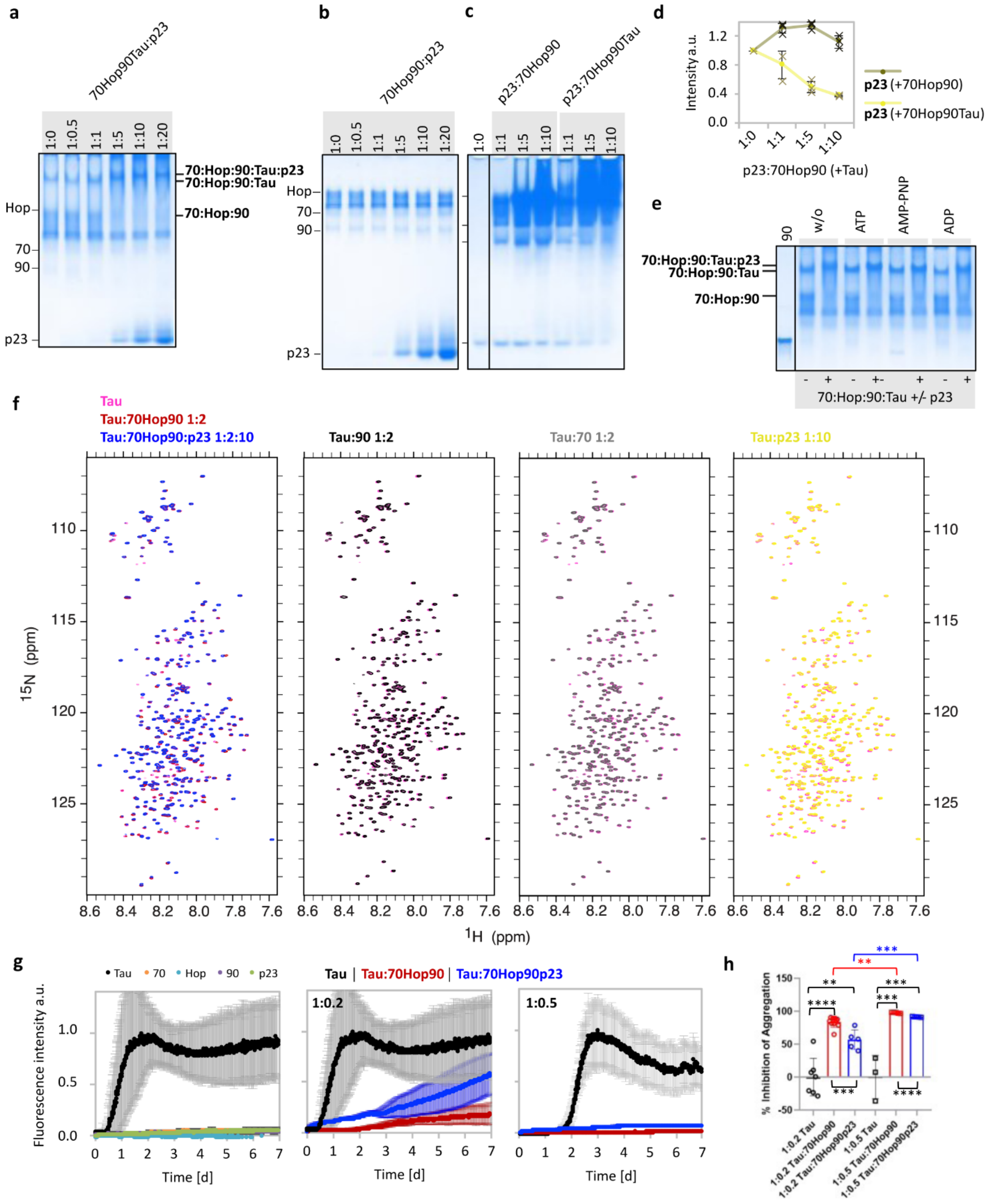

Supplementary Fig. 2. Tau promotes p23 binding to the Hsp70/Hsp90 multichaperone complex (related to Fig. 2). A) Native PAGE demonstrating the formation of the 5-component Hsp70:Hop:Hsp90:Tau:p23 complex. Hsp70 and Hsp90 are abbreviated as “70” and “90”, respectively. B-C) Native PAGE indicating that p23 only interacts stably with the Hsp70:Hop:Hsp90 complex in the presence of Tau (Hsp70:Hop:Hsp90:Tau molar ratio of 1:1:1:5). D) Quantitative analysis of the free p23 band from (c). Data are presented as mean +/ SD from three independent experiments. E) Native PAGE demonstrating the formation of a stable Hsp70:Hop:Hsp90:Tau:p23 complex (mixed in a molar ratio of 1:1:1:5:5) independent of the presence of nucleotides; (-) and (+) indicate the absence and presence of p23, respectively. F) 2D ^{15}N-^{1}H HSQC spectra of Tau alone (pink) and in presence of the Hsp70:Hop:Hsp90 complex (molar ratio

of 1:1:1) without (red) and with p23 (blue), Hsp90 (black), Hsp70 (dark grey) and p23 alone (yellow). Tau:chaperone molar ratios are indicated on top of each spectrum. G) Left panel: aggregation assay of the individual proteins Hsp70, Hop, Hsp90, Tau and p23 showing aggregation for Tau only. Middle and right panel: aggregation assay of Tau alone and in presence of the Hsp70/Hsp90 chaperone machinery. At a Tau:machinery molar ratio of 1:0.2 the chaperone complex starts to lose its inhibitory activity after ~2.5 d (middle panel). Using a higher Tau:machinery molar ratio of 1:0.5 inhibits Tau aggregation for the time course of the experiment (right panel). For panels g-h, data are presented as mean values +/- SD from n independent experiments. For individual proteins, n=7 for Tau, n=5 for Hsp70, n=5 for Hop, n=5 for Hsp90, and n=5 for p23. For 1:0.2 mole ratios, n=7 for Tau, n=10 for Tau:Hsp70:Hop:Hsp90, and n=5 for Tau:Hsp70:Hop:Hsp90:p23. For 1:0.5 mole ratios, n=3 for Tau, n=5 for Tau:Hsp70:Hop:Hsp90, and n=5 for Tau:Hsp70:Hop:Hsp90:p23. H) Inhibition of Tau aggregation (from panel g) depends on the dose of Hsp70:Hop:Hsp90 machinery. The addition of p23 slightly lowers the inhibitory effect of Hsp70:Hop:Hsp90 towards Tau aggregation. Null hypothesis testing was performed using an unpaired, two-tailed t test. (* $p \leq 0.033$, ** $p \leq 0.0021$, *** $p \leq 0.0002$, **** $p \leq 0.0001$).

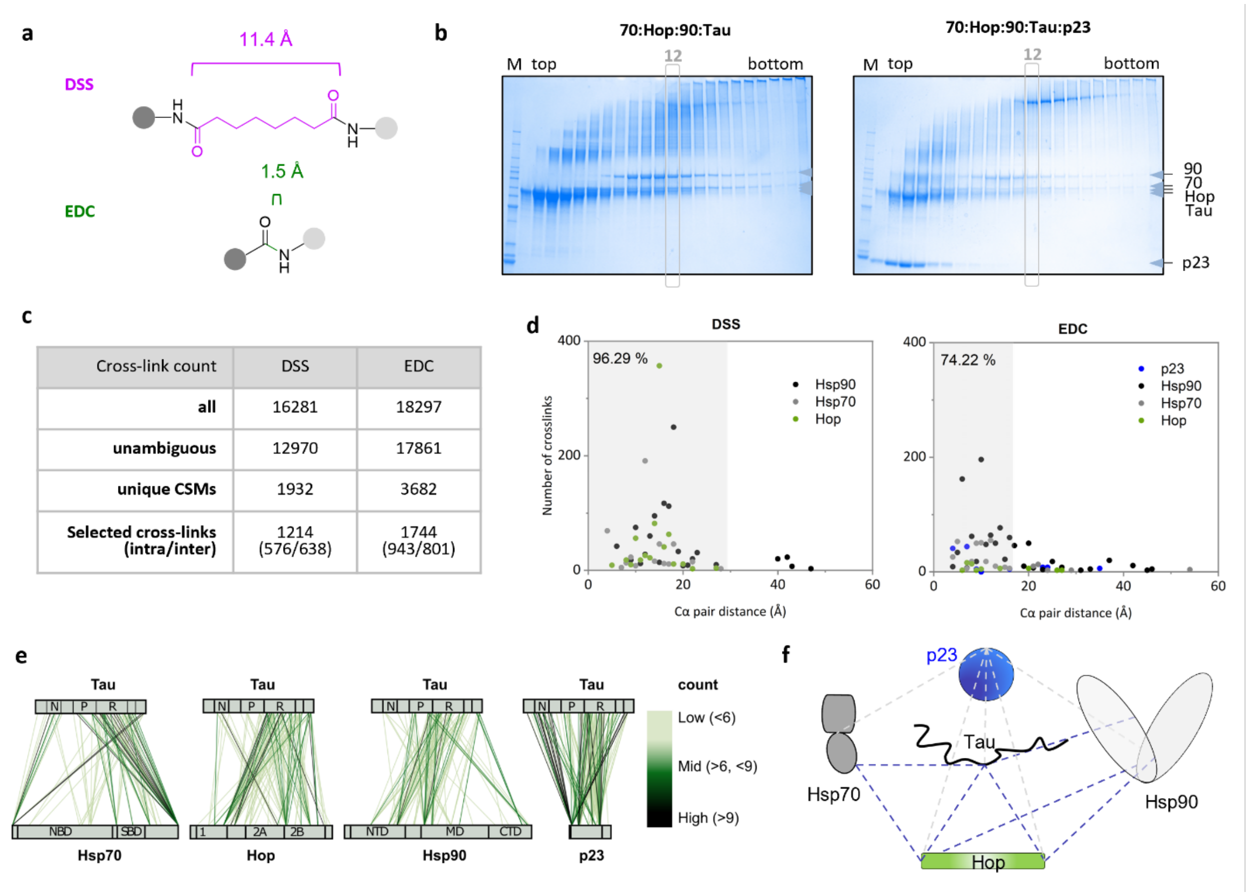

Cross-link count	DSS	EDC
**all**	16281	18297
**unambiguous**	12970	17861
**unique CSMs**	1932	3682
**Selected cross-links (intra/inter)**	1214 (576/638)	1744 (943/801)

Supplementary Fig. 3. Chemical crosslinking of the Hsp70:Hop:Hsp90:Tau:p23 complex shows similar protein arrangement using two different crosslinkers (related to Fig. 4). A) Crosslinker arm of DSS (crosslinking primary amines) and EDC (crosslinking carboxylic acids with primary amines). B) After DSS-crosslinking, the distribution of complexes subjected to sucrose gradient ultracentrifugation (using 10%-25% sucrose from the top to the bottom of the gradient) are visualized in SDS PAGE with a 4-15% acrylamide gradient gel. The fractions boxed in grey were used for SEC and MS experiments. Grey arrows indicate the positions of non-crosslinked Hsp70 ("70"), Hop, Hsp90 ("90"), Tau, and p23. C) The number of detected crosslinks is listed by unambiguously identified crosslinks, unique crosslink spectrum matches (CSMs) and selected crosslinks based on a set threshold of at least three CSMs. D) Distance measurement of intramolecular crosslinks detected within Hsp70, Hop, Hsp90 and p23 using the structures deposited in the PDB: 5fwk (Hsp90),[1] 5aqz (Hsp70-NBD),[2] 4po2 (Hsp70-SBD),[3] 1elw (Hop-TPR1),[4] 1elr (Hop-TPR2A)[4] and 1ejf (p23).[5] Up to 96.29 % for DSS and 74.22 % for EDC of the analyzed crosslinks were identified as valid (grey shaded). E) EDC crosslink analysis between two proteins within the Hsp70:Hop:Hsp90:Tau:p23 complex color coded based on the number of crosslinked peptide spectrum matches (CSMs). Count of CSM higher than 9 (dark green), 6-9 (green) and 3-6 (light green). Protein domains are indicated as grey bars; Hsp70: NBD-linker-SBD; Hop: TPR1-DP1-TPR2A-TPR2B-DP2; Hsp90: NTD-cl-MD-CTD; Tau: N1-N2-P1-P2-R1-R2-R3-R4-R'; p23: NTD-tail. F) The major intermolecular crosslinks are illustrated connecting the repeat domain of Tau with Hsp70's SBD, Hsp90's NTD and MD, the TPR1 and TPR2B domains of Hop and p23.

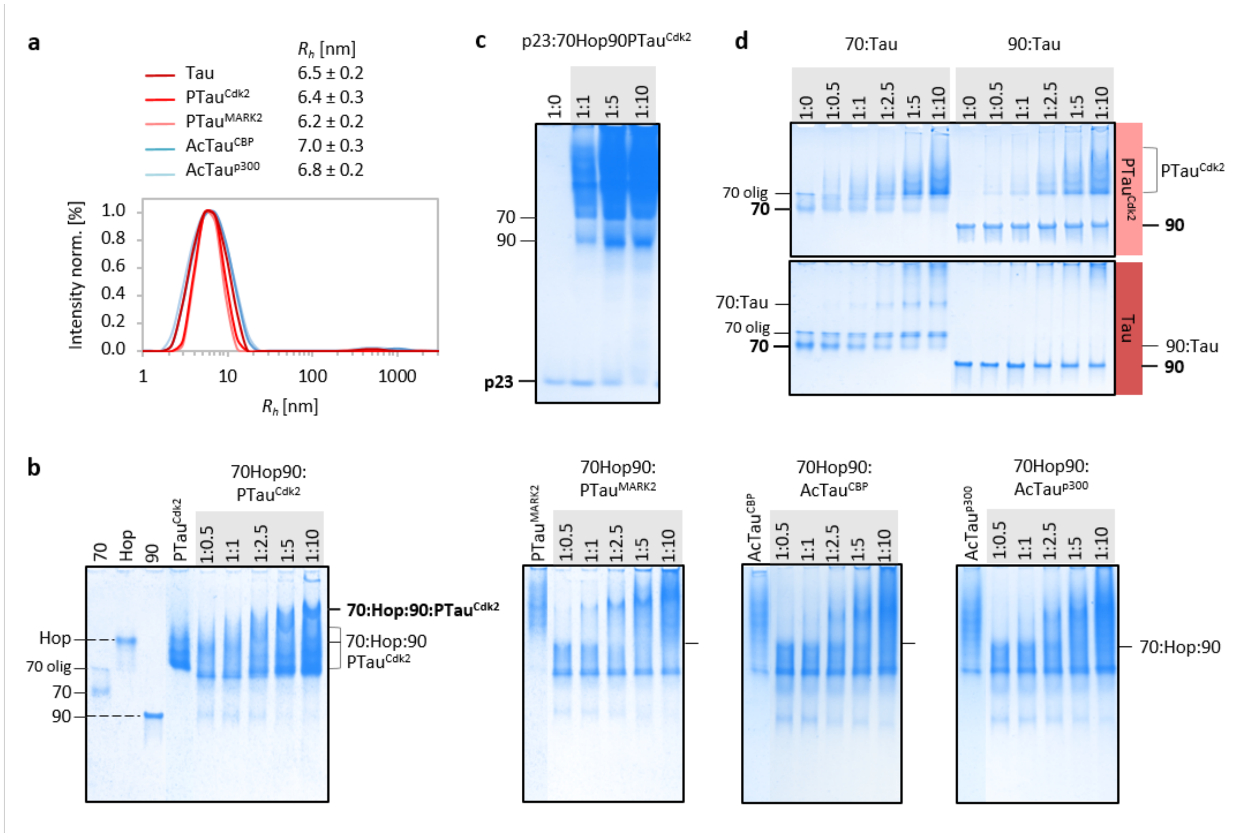

Supplementary Fig. 4. The Hsp70/Hsp90 chaperone machinery interacts with phosphorylated as well as acetylated Tau (related to Fig. 6). Hsp70 and Hsp90 are abbreviated as “70” and “90”, respectively. A) DLS analysis showing the hydrodynamic radii of unmodified, acetylated (AcTau) and phosphorylated (PTau) Tau using the kinases Cdk2 and MARK2 and the acetyltransferases CBP and p300. No oligomerization was observed upon Tau modification. Errors represent SD from three measurements. B) Interaction between modified Tau and the Hsp70/Hsp90 chaperone machinery monitored by native PAGE. An up to 10-fold excess of the modified Tau proteins characterized in (A) were used. C) Attenuation of the p23 native PAGE band at increasing concentrations of the Hsp70:Hop:Hsp90:PTauCdk2 complex. D) Native PAGE analysis of Hsp70 (left) and Hsp90 (right) in the presence of increasing concentrations of PTauCdk2 (top) and unmodified Tau (bottom).

Scanned Gel Images Related to Supplementary Figure S1

Figure S1a

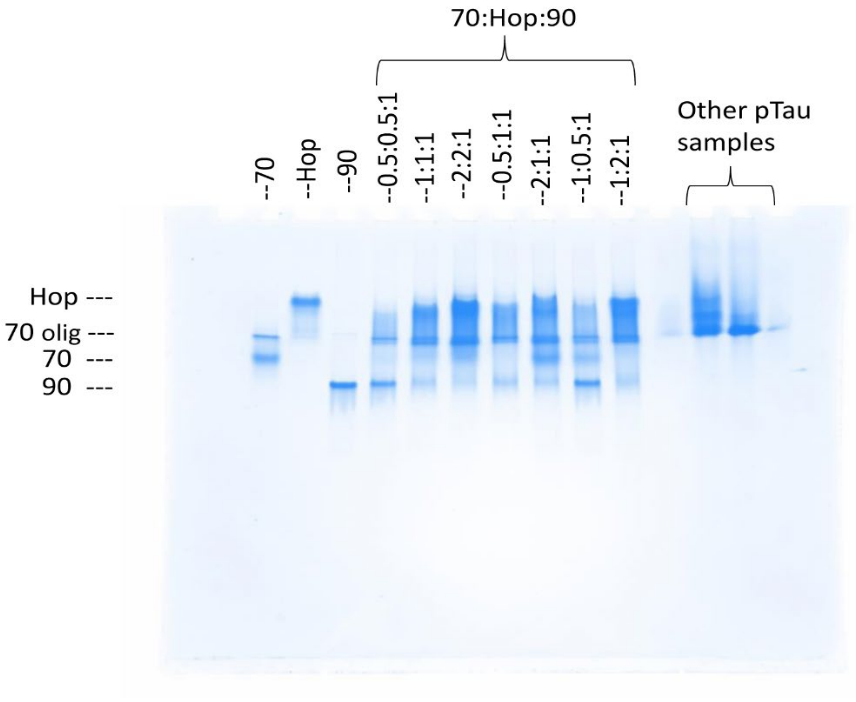

Figure S1d

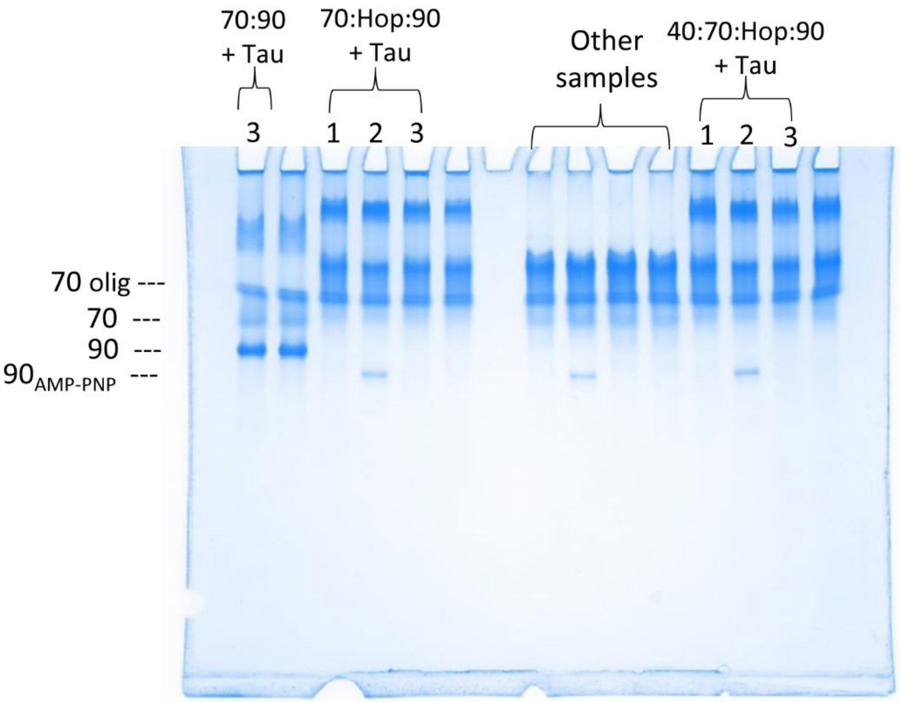

70:90
+ Tau
3
70:Hop:90
+ Tau
1 2 3
Other
samples
40:70:Hop:90
+ Tau
1 2 3
70 olig ---
70 ---
90 ---
90AMP-PNP ---

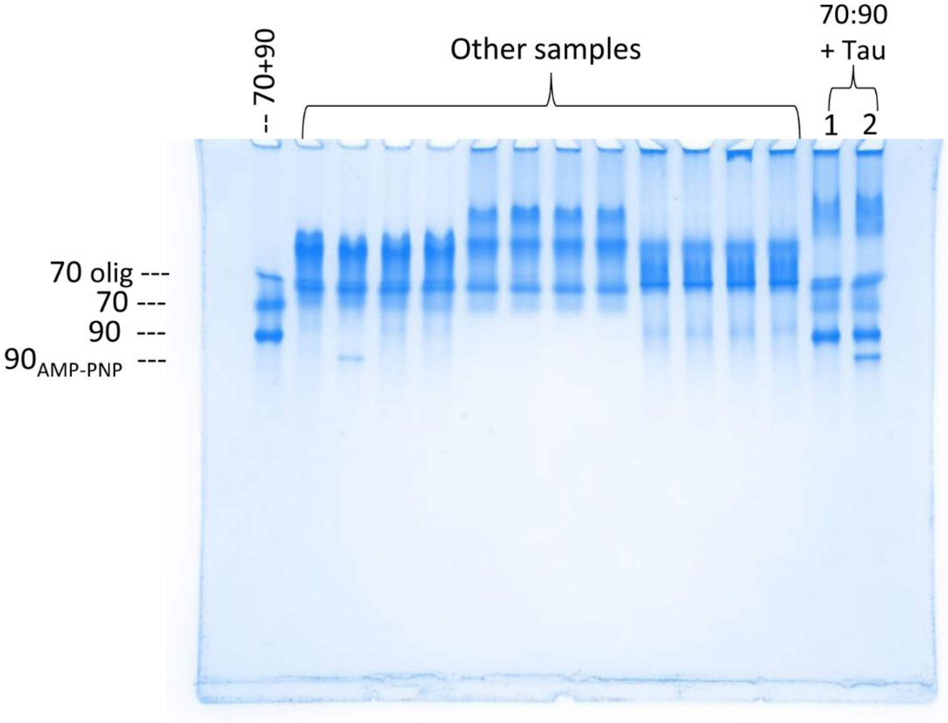

-- 70+90
Other samples
70:90
+ Tau
1 2
70 olig ---
70 ---
90 ---
90AMP-PNP ---

Figure S2a

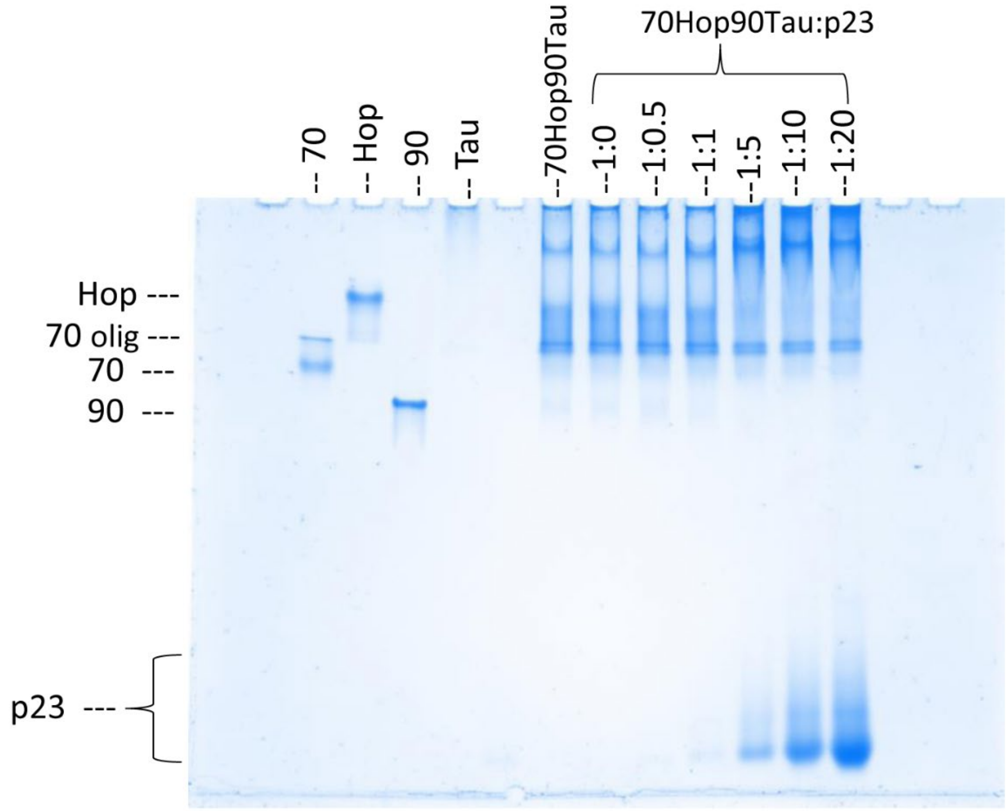

Figure S2b

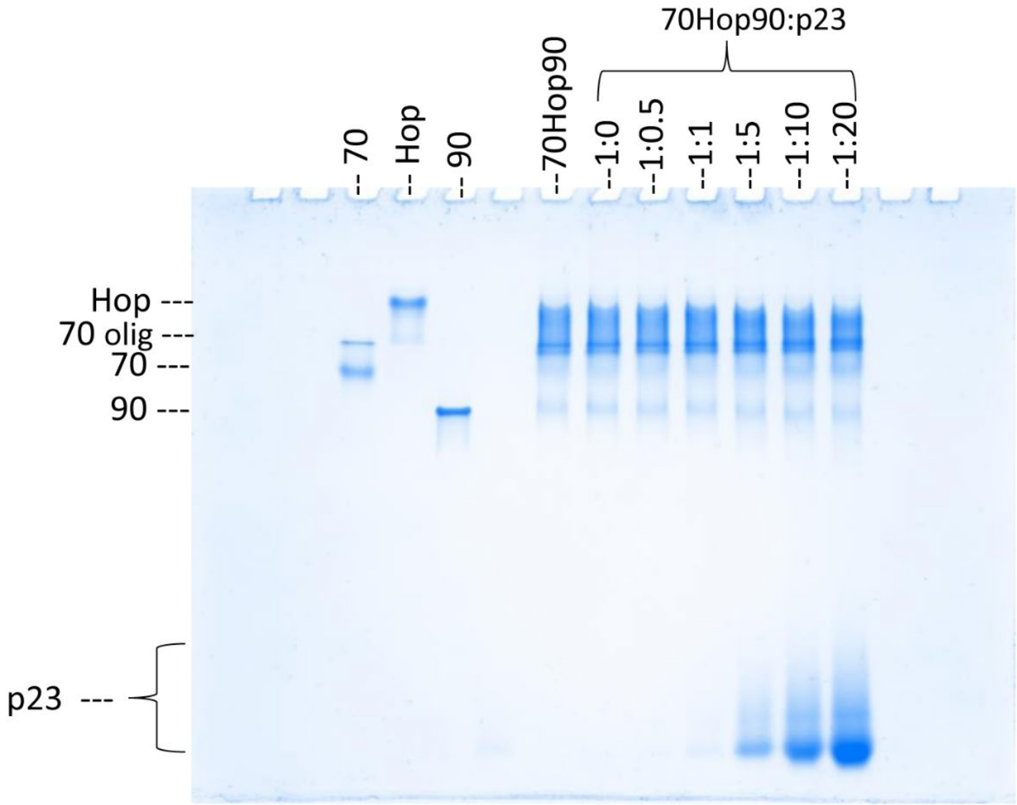

Figure S2c

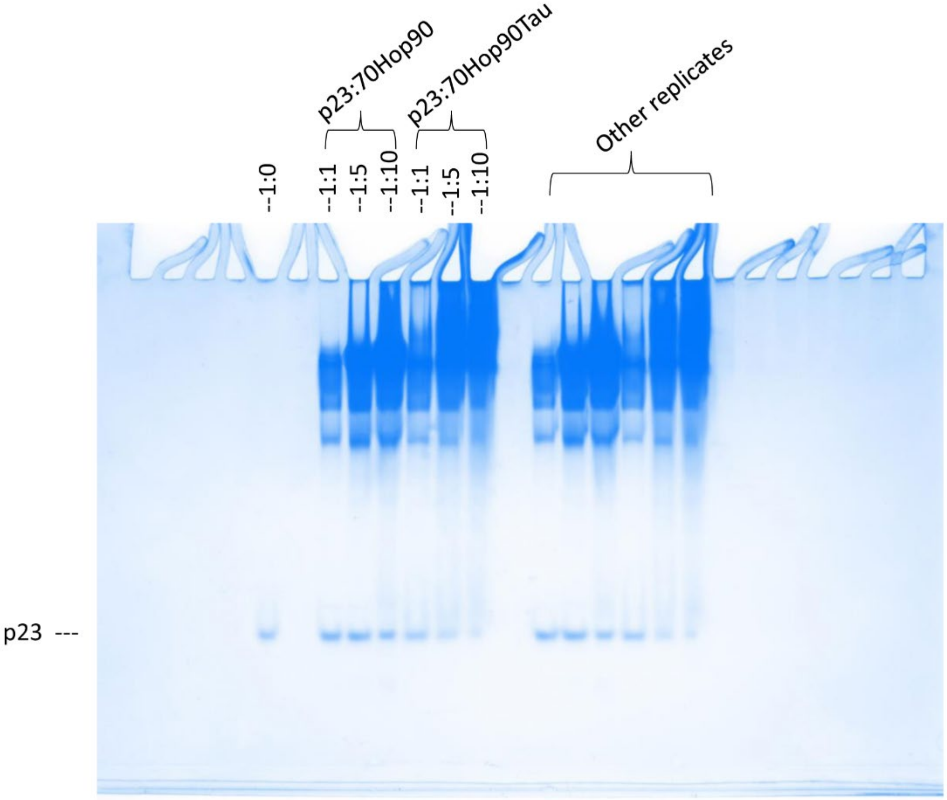

Figure S2e

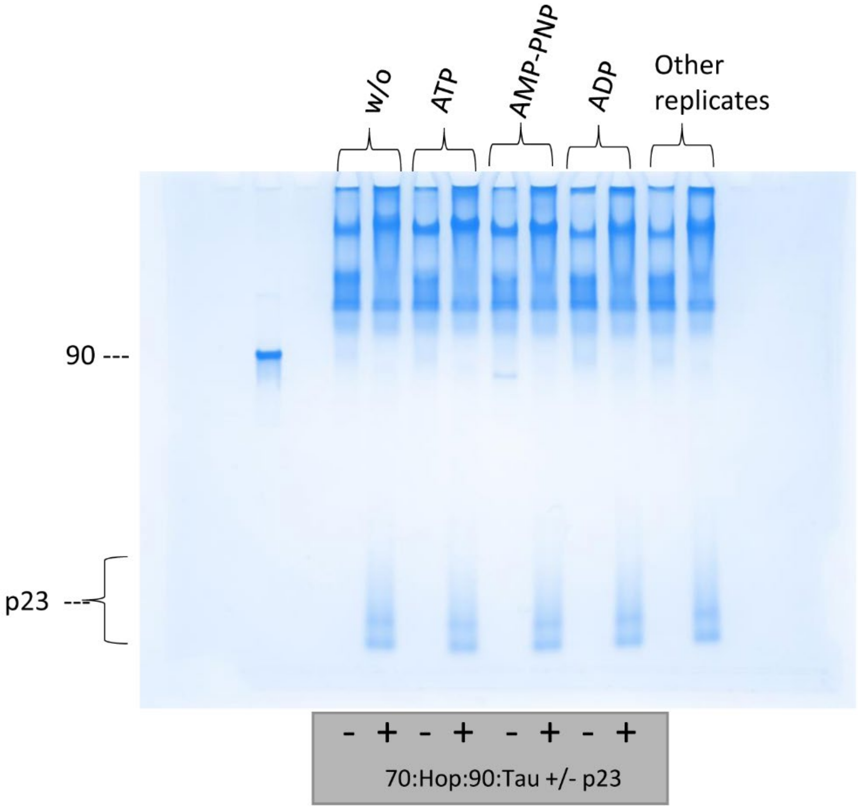

Figure S3b

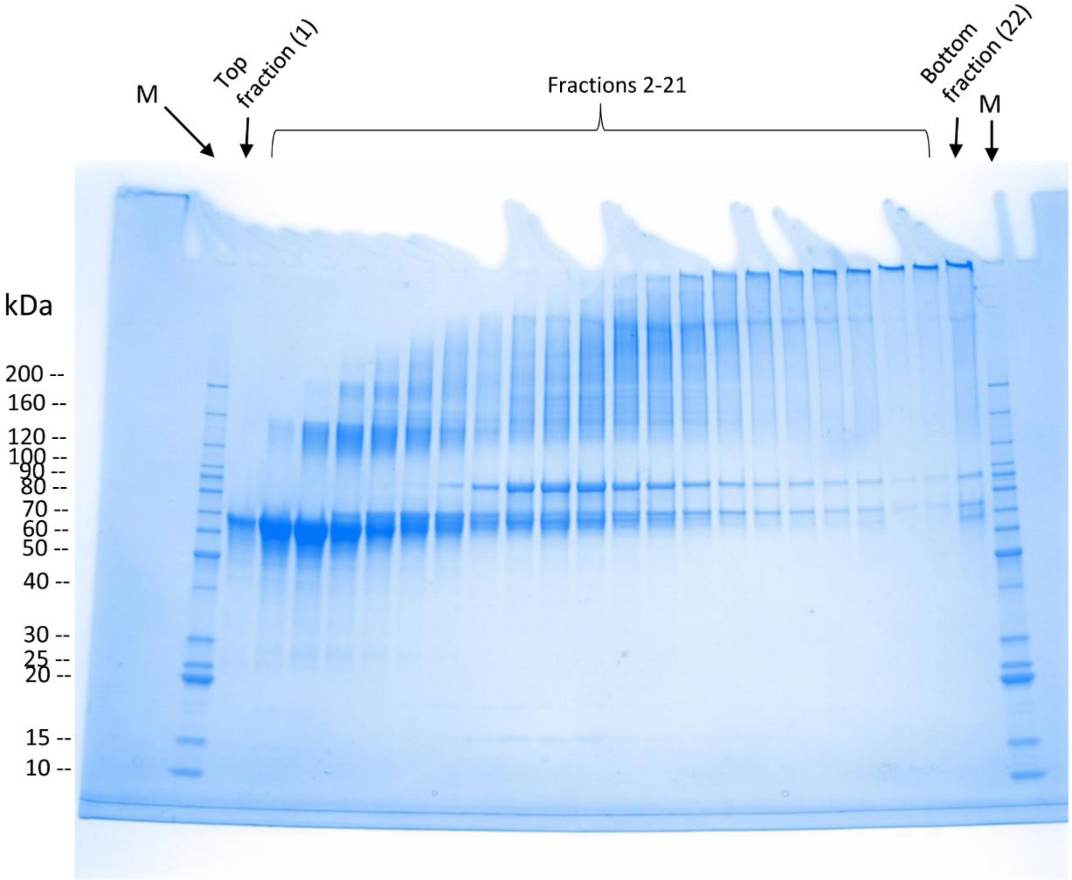
M
Top fraction (1)
Fractions 2-21
Bottom fraction (22)
M
kDa
200 --
160 --
120 --
100 --
90 --
80 --
70 --
60 --
50 --
40 --
30 --
25 --
20 --
15 --
10 --

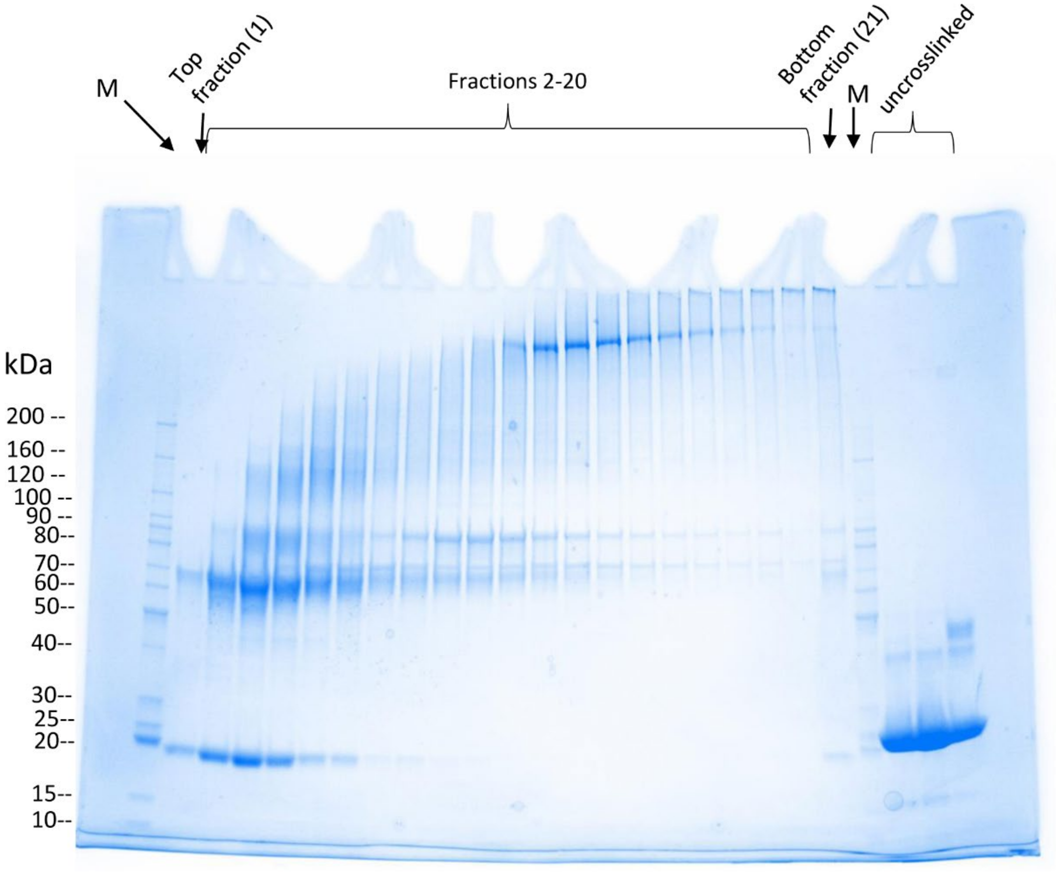
M
Top fraction (1)
Fractions 2-20
Bottom fraction (21)
M
uncrosslinked
kDa
200 --
160 --
120 --
100 --
90 --
80--
70--
60--
50--
40--
30--
25--
20--
15--
10--

Figure S4b

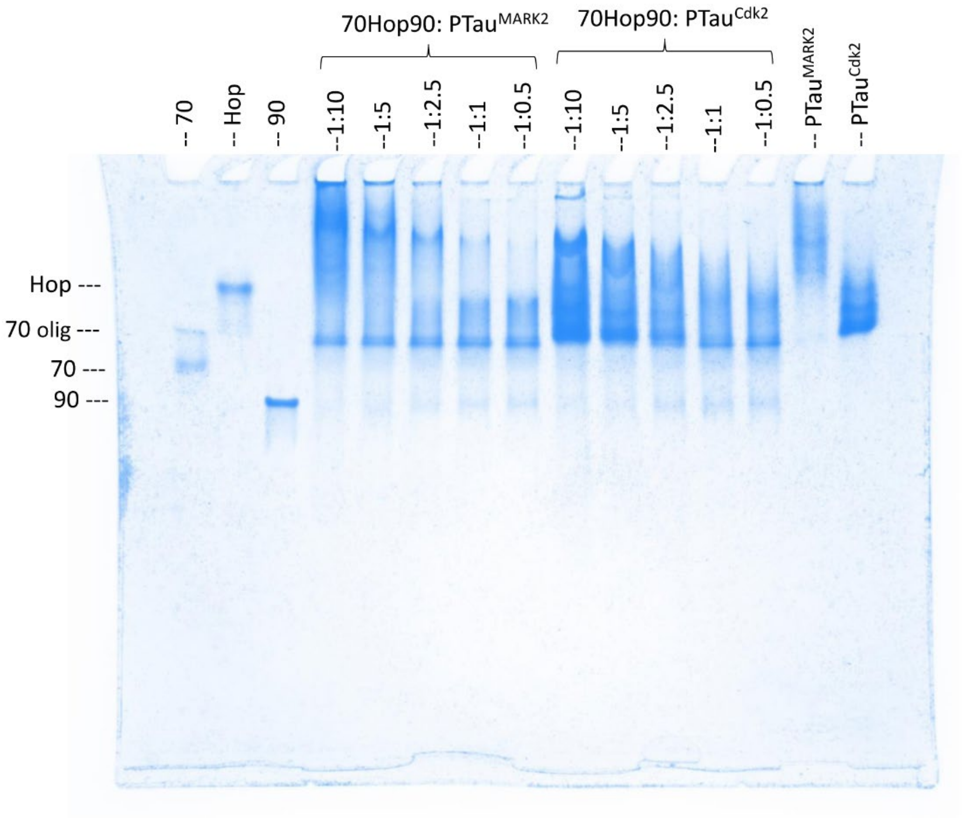
70Hop90: PTau^MARK2
70Hop90: PTau^Cdk2
-- 70
-- Hop
-- 90
--1:10
--1:5
--1:2.5
--1:1
--1:0.5
--1:10
--1:5
--1:2.5
--1:1
--1:0.5
-- PTau^MARK2
-- PTau^Cdk2
Hop ---
70 olig ---
70 ---
90 ---

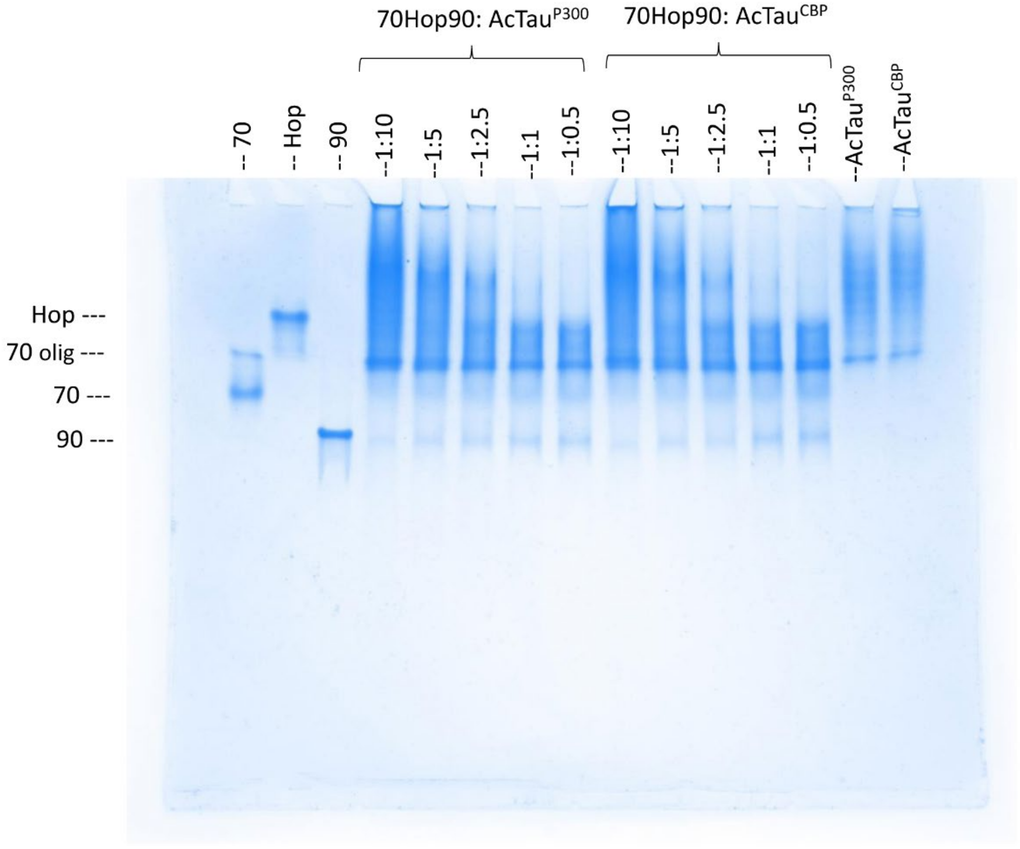
70Hop90: AcTau^P300
70Hop90: AcTau^CBP
-- 70
-- Hop
-- 90
--1:10
--1:5
--1:2.5
--1:1
--1:0.5
--1:10
--1:5
--1:2.5
--1:1
--1:0.5
--AcTau^P300
--AcTau^CBP
Hop ---
70 olig ---
70 ---
90 ---

Figure S4c

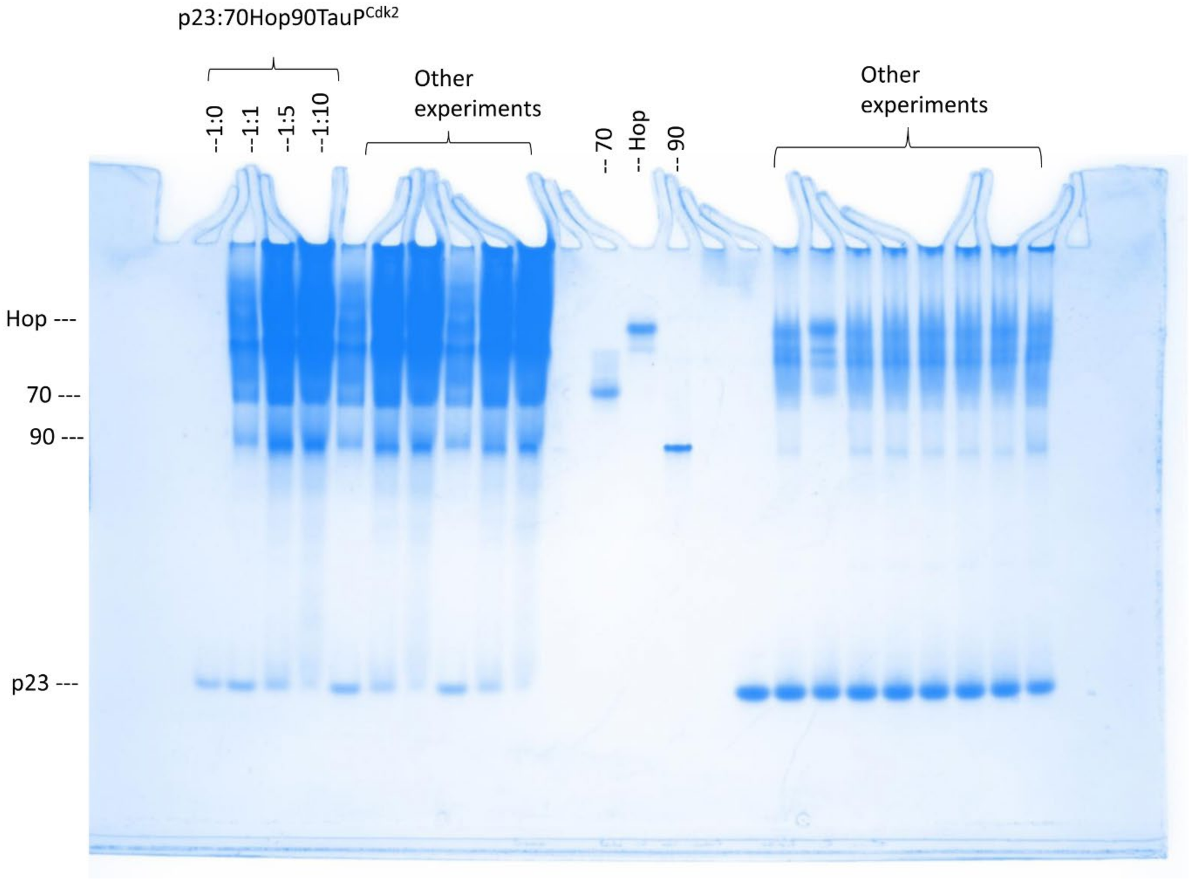

Figure S4d

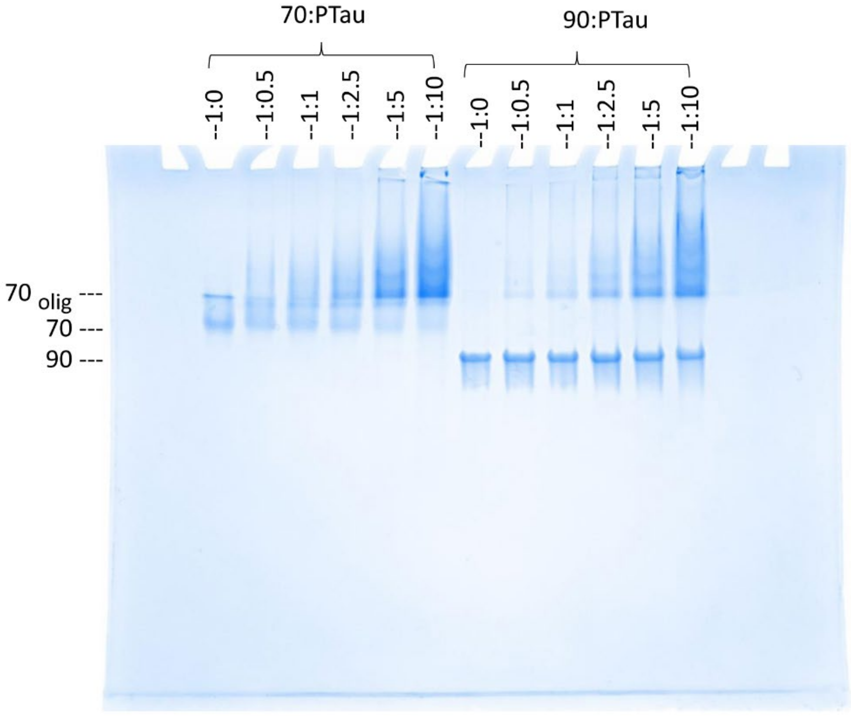

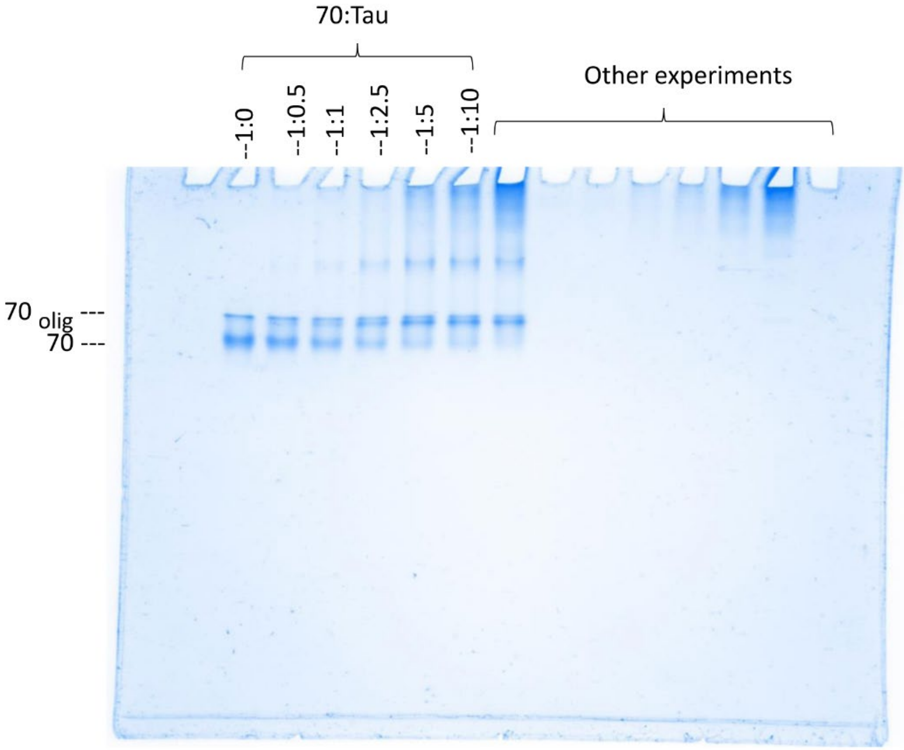
70:Tau
--1:0
--1:0.5
--1:1
--1:2.5
--1:5
--1:10
Other experiments
70 olig ---
70 ---

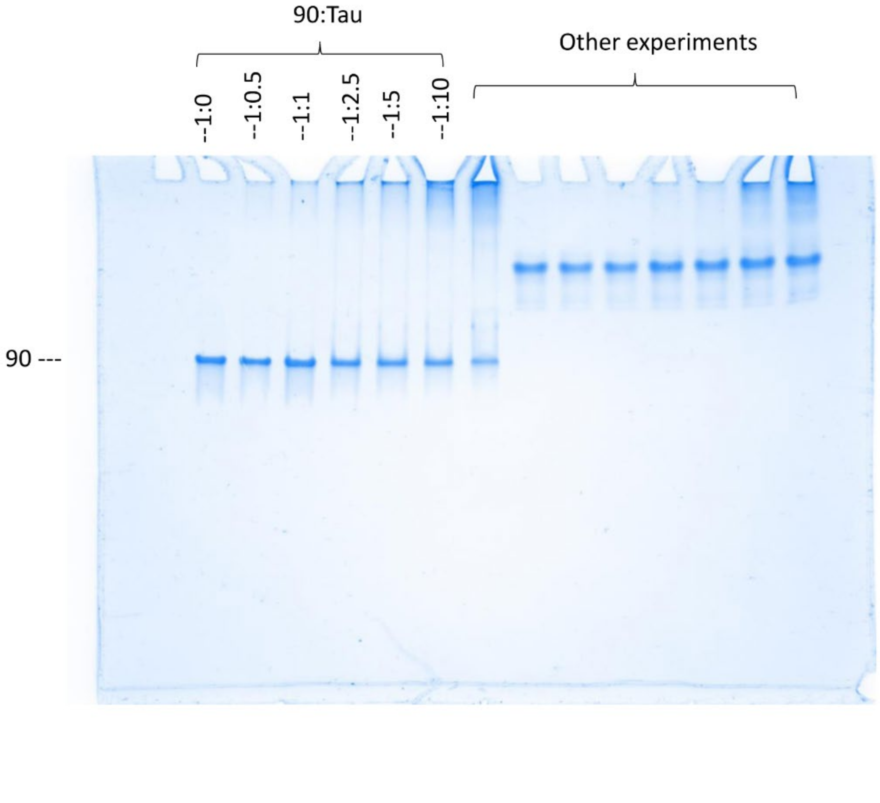
90:Tau
--1:0
--1:0.5
--1:1
--1:2.5
--1:5
--1:10
Other experiments
90 ---

Supplementary Tables

Supplementary Table 1. MaxQuant (MQ) intensity-based absolute quantification (iBAQ) values referring to protein abundance in the Hsp70:Hop:Hsp90, Hsp70:Hop:Hsp90:Tau and the Hsp70:Hop:Hsp90:Tau:p23 complexes. Corresponding results for the bands of the individual proteins of the Hsp70/Hsp90 chaperone machinery are shown for comparison. Hsp70 and Hsp90 are abbreviated as “70” and “90”, respectively.

**band cut**	**70**	**Hop**	**90**	**70 Hop 90**	**70 Hop 90 Tau**	**70 Hop 90 Tau p23**
**protein identified**						
**Hsp70**	4,62E+09	1,85E+07	2,25E+06	5,91E+09	2,19E+09	4,17E+09
**Hop**	4,88E+04	3,40E+09	3,42E+06	1,36E+10	2,84E+09	6,20E+09
**Hsp90**	1,93E+06	1,57E+06	8,67E+09	1,28E+10	3,88E+09	1,14E+10
**Tau**	2,90E+07	1,89E+07	1,00E+07	8,27E+06	2,04E+09	1,19E+10
**p23**						5,74E+08

Supplementary Table 2. In vitro Tau phosphorylation sites using Cdk2 kinase detected by mass spectrometry.

#Phosphorylation site	Intensity	Localization probability
T175	27.63	1
T181	25.30	0.999
S199	26.78	0.922
S202	22.27	0.724
T205	30.37	0.999
T205	26.78	0.999
T217	23.45	0.936
T220	23.74	0.724
T231	24.14	0.999
S235	24.14	0.969
T245	26.47	1
S262	27.20	0.971
T263	27.82	0.991
S396	24.58	1
S400	23.36	0.815
T403	25.05	0.581
S404	24.13	0.911
S422	24.22	0.999
S435	21.79	0.839

References

1. Verba, K. A. et al. Atomic structure of Hsp90-Cdc37-Cdk4 reveals that Hsp90 traps and stabilizes an unfolded kinase. Science **352**, 1542-1547, doi:10.1126/science.aaf5023 (2016).
2. Cheeseman, M. D. et al. Exploiting Protein Conformational Change to Optimize Adenosine-Derived Inhibitors of HSP70. J Med Chem **59**, 4625-4636, doi:10.1021/acs.jmedchem.5b02001 (2016).
3. Zhang, P., Leu, J. I., Murphy, M. E., George, D. L. & Marmorstein, R. Crystal structure of the stress-inducible human heat shock protein 70 substrate-binding domain in complex with peptide substrate. PLoS One **9**, e103518, doi:10.1371/journal.pone.0103518 (2014).
4. Scheufler, C. et al. Structure of TPR domain-peptide complexes: critical elements in the assembly of the Hsp70-Hsp90 multichaperone machine. Cell **101**, 199-210, doi:10.1016/S0092-8674(00)80830-2 (2000).
5. Weaver, A. J., Sullivan, W. P., Felts, S. J., Owen, B. A. & Toft, D. O. Crystal structure and activity of human p23, a heat shock protein 90 co-chaperone. J Biol Chem **275**, 23045-23052, doi:10.1074/jbc.M003410200 (2000).
