# Supplementary material for: Hsp multichaperone complex buffers pathologically modified Tau
Source: Nat Commun. 2022 Jun 27;13:3668. doi: 10.1038/s41467-022-31396-z (PMC9237115; doi:10.1038/s41467-022-31396-z)
Supplement: Supplementary file 3 — Description of Additional Supplementary Files [file 41467_2022_31396_MOESM3_ESM.pdf]

Description of Additional Supplementary Files

File Name: Supplementary Data 1
Description: Selected intra- and intermolecular crosslinks within the Hsp70:Hop:Hsp90:Tau:p23 complex crosslinked with disuccinimidyl suberate (DSS).

File Name: Supplementary Data 2
Description: Selected intra- and intermolecular crosslinks within the Hsp70:Hop:Hsp90:Tau:p23 complex crosslinked with 1-ethyl-3-(3- dimethylaminopropyl) carbodiimide (EDC).
