# Supplementary material for: Hsp multichaperone complex buffers pathologically modified Tau
Source: Nat Commun. 2022 Jun 27;13:3668. doi: 10.1038/s41467-022-31396-z (PMC9237115; doi:10.1038/s41467-022-31396-z)
Supplement: Supplementary file 6 — Reporting Summary [file 41467_2022_31396_MOESM6_ESM.pdf]

nature research

Corresponding author(s): Markus Zweckstetter

Last updated by author(s): Jun 11, 2022

Reporting Summary

Nature Research wishes to improve the reproducibility of the work that we publish. This form provides structure for consistency and transparency in reporting. For further information on Nature Research policies, see our Editorial Policies and the Editorial Policy Checklist.

Statistics

For all statistical analyses, confirm that the following items are present in the figure legend, table legend, main text, or Methods section.

n/a	Confirmed	
☐	☒	The exact sample size (n) for each experimental group/condition, given as a discrete number and unit of measurement
☐	☒	A statement on whether measurements were taken from distinct samples or whether the same sample was measured repeatedly
☐	☒	The statistical test(s) used AND whether they are one- or two-sided<br>*Only common tests should be described solely by name; describe more complex techniques in the Methods section.*
☒	☐	A description of all covariates tested
☒	☐	A description of any assumptions or corrections, such as tests of normality and adjustment for multiple comparisons
☐	☒	A full description of the statistical parameters including central tendency (e.g. means) or other basic estimates (e.g. regression coefficient) AND variation (e.g. standard deviation) or associated estimates of uncertainty (e.g. confidence intervals)
☐	☒	For null hypothesis testing, the test statistic (e.g. F, t, r) with confidence intervals, effect sizes, degrees of freedom and P value noted<br>*Give P values as exact values whenever suitable.*
☒	☐	For Bayesian analysis, information on the choice of priors and Markov chain Monte Carlo settings
☒	☐	For hierarchical and complex designs, identification of the appropriate level for tests and full reporting of outcomes
☒	☐	Estimates of effect sizes (e.g. Cohen's d, Pearson's r), indicating how they were calculated

Our web collection on statistics for biologists contains articles on many of the points above.

Software and code

Policy information about availability of computer code

Data collection	NMR data were acquired using Topspin 3.5pl5; ThT fluorescence data were collected using Spark control software v2.2 by Tecan; Trp fluorescence data were collected using Cary Eclipse Scan Application v1.2(147); MS data were collected with Xcalibur v4.4; SEC chromatograms were collected with the UNICORN software v7.2; DLS data were acquired with DYNAMICS v7.10.0.23.
Data analysis	NMR data were processed with Topspin 3.6.1 and analyzed using NMRFAM-Sparky v1.4 powered by Sparky v3.1. UCSF Chimera v1.14 was used to analyze intramolecular cross-links. The pLink software v2.3.9 was used to analyze the MS data, the xiView Webserver was used to creat network plots showing cross-links. Band intensities were quantified with ImageJ v1.52n and v1.53e. DLS data were analyzed with DYNAMICS v7.10.0.23. Microsoft Excel v16.43 and Graphpad Prism v8.0.1 were used for fitting data as well as for statistical analysis.

For manuscripts utilizing custom algorithms or software that are central to the research but not yet described in published literature, software must be made available to editors and reviewers. We strongly encourage code deposition in a community repository (e.g. GitHub). See the Nature Research guidelines for submitting code & software for further information.

Data

Policy information about availability of data

All manuscripts must include a data availability statement. This statement should provide the following information, where applicable:

- Accession codes, unique identifiers, or web links for publicly available datasets
- A list of figures that have associated raw data
- A description of any restrictions on data availability

All MS raw files were deposited to the ProteomeXchange Consortium (HYPERLINK "http://www.proteomexchange.org/" www.proteomexchange.org) via the PRIDE1 partner repository with the dataset identifier PXD032037.58 All PDB codes cited (“5fwk” http://doi.org/ HYPERLINK "http://doi.org/10.2210/pdb5FWK/pdb"10.2210/pdb5FWK/pdb, “5aqz” http://doi.org/ HYPERLINK "http://doi.org/10.2210/pdb5AQZ/pdb"10.2210/pdb5AQZ/pdb, “4po2” http://doi.org/ HYPERLINK

"http://doi.org/10.2210/pdb4PO2/pdb"10.2210/pdb4PO2/pdb, “1elw” http://doi.org/ HYPERLINK "http://doi.org/10.2210/pdb1ELW/pdb"10.2210/pdb1ELW/pdb, “1elr” http://doi.org/ HYPERLINK "http://doi.org/10.2210/pdb1ELR/pdb"10.2210/pdb1ELR/pdb, “1ejf” http://doi.org/ HYPERLINK "http://doi.org/10.2210/pdb1EJF/pdb"10.2210/pdb1EJF/pdb)63-67 are publicly available in the PDB. Source data are provided with this paper.

Field-specific reporting

Please select the one below that is the best fit for your research. If you are not sure, read the appropriate sections before making your selection.

- [x] Life sciences
- [ ] Behavioural & social sciences
- [ ] Ecological, evolutionary & environmental sciences

For a reference copy of the document with all sections, see nature.com/documents/nr-reporting-summary-flat.pdf

Life sciences study design

All studies must disclose on these points even when the disclosure is negative.

Sample size	No calculations were performed to predetermine sample size.<br>For aggregation experiments at least 3 samples were used to occupy different spots in the microwell plate to account for possible fluctuations in temperature and agitation. In vitro complex reconstitutions used a minimum of 3 samples, and from different batches of proteins to account for protein batch-to-batch variation. The affinity determination by gels and tryptophan quenching was semiquantitative and thus performed using a minimum of n=2, with final KD values regarded as apparent. MW determination was also semiquantitative, and thus only used 2 samples per condition. NMR data reported for intensity ratios and chemical shift perturbations come from one representative spectrum (per sample condition) and error bars were taken from signal-to-noise since the S/N per peak in an NMR spectrum is an indication of the variability of the intensity quantification and measurement of the chemical shift.
Data exclusions	No data have been excluded while reporting this study.
Replication	MW size determination by size exclusion chromatography used 2 replicates.<br>NMR: One representative spectrum was used for each data set reported here for the reason explained above.<br>Data were reproducible in these experiments - all replicates were successful.<br>Mass spectrometry was performed once per cross-linking condition (EDC or DSS), and each analysis used at least 8 peptide fractions. Data from two cross-linking strategies complemented each other.
Randomization	Randomization in terms of randomized sampling was not relevant because we did not select samples meant to represent larger populations. However randomization was used in the sense that different batches of proteins with random batch combinations were used for performing protein-protein interaction experiments. In the case of aggregation assays, random locations in the plate reader were also used per sample condition to eliminate bias from possible systematic differences in temperature, humidity, and agitation in the plate reader.
Blinding	Blinding was not relevant to this study, because decision-making has no impact on the experiment and there is no risk of bias.

Reporting for specific materials, systems and methods

We require information from authors about some types of materials, experimental systems and methods used in many studies. Here, indicate whether each material, system or method listed is relevant to your study. If you are not sure if a list item applies to your research, read the appropriate section before selecting a response.

Materials & experimental systems

n/a	Involved in the study
x	Antibodies
x	Eukaryotic cell lines
x	Palaeontology and archaeology
x	Animals and other organisms
x	Human research participants
x	Clinical data
x	Dual use research of concern

Methods

n/a	Involved in the study
x	ChIP-seq
x	Flow cytometry
x	MRI-based neuroimaging
